# Satisfaction with social connectedness as a predictor for positive and negative symptoms of psychosis: A PHAMOUS study

**DOI:** 10.1192/j.eurpsy.2022.329

**Published:** 2022-09-01

**Authors:** J.S. Vogel, J. Bruins, S. De Jong, R. Knegtering, A.A. Bartels-Velthuis, M. Van Der Gaag, S. Castelein

**Affiliations:** 1 University of Groningen, Clinical Psychology, Groningen, Netherlands; 2 Lentis Psychiatric Institute, Lentis Research, Groningen, Netherlands; 3 University of Groningen, University Medical Center Groningen, University Center For Psychiatry, Rob Giel Research Center, Groningen, Netherlands; 4 VU University Amsterdam, Department Of Clinical Psychology, Amsterdam, Netherlands

**Keywords:** social connectedness, PSYCHOTIC DISORDERS, positive symptoms, negative symptoms

## Abstract

**Introduction:**

Social connectedness might positively influence the course of clinical symptoms in people with psychotic disorders.

**Objectives:**

This study examines satisfaction with social connectedness (SSC) as predictor of positive and negative symptoms in people with a psychotic disorder.

**Methods:**

Data from the Pharmacotherapy Monitoring and Outcome Survey (PHAMOUS, 2014-2019) was used from patients diagnosed with a psychotic disorder (N=2109). Items about social connectedness of the Manchester short assessment of Quality of Life (ManSA) were used to measure SSC. Linear mixed models were used to estimate the association of SSC with the Positive and Negative Syndrome Scale (PANSS) after one and two years against α=0.01. Analyses were adjusted for symptoms, time since onset, gender and age. Additionally, fluctuation of positive and negative symptom scores over time was estimated.

**Results:**

Mean duration of illness of the sample was 18.8 years (SD 10.7) with >65% showing only small variation in positive and negative symptoms over a two to five-year time period. After adjustment for covariates, SSC showed to be negatively associated with positive symptoms after one year (β=-0.47, p<0.001, 95% CI=-0.70,-0.25) and two years (β =-0.59, p<0.001, 95% CI = -0.88,-0.30), and for negative symptoms after one year (β=-0.52, p<0.001, 95% CI = -0.77,-0.27). The prediction of negative symptoms was not significant at two years.

**Conclusions:**

This research indicates that interventions on SSC might positively impact mental health for people with psychosis. SSC is a small and robust predictor of future levels of positive symptoms. Negative symptoms could be predicted by SSC at one year.

**Disclosure:**

No significant relationships.

